# Adaptive Information Visualization for Maritime Traffic Stream Sensor Data with Parallel Context Acquisition and Machine Learning

**DOI:** 10.3390/s19235273

**Published:** 2019-11-29

**Authors:** Kwang-il Kim, Keon Myung Lee

**Affiliations:** 1 Department of Marine Industry and Maritime Police, Jeju National University, Jeju 64343, Korea; kki@jejunu.ac.kr; 2 Dept. of Computer Science, Chungbuk National University, Cheongju 28644, Korea

**Keywords:** big data, stream data, context-aware service, distributed and parallel processing, vessel traffic service, maritime traffic stream sensor data

## Abstract

Excessive information significantly increases the mental burden on operators of critical monitoring services such as maritime and air traffic control. In these fields, vessels and aircraft have sensors that transmit data to a control center. Because of the large volume of collected data, it is infeasible for monitoring stations to display all of the information on monitoring screens that have limited sizes. This paper proposes a method for automatically selecting maritime traffic stream data for display from a large number of candidates in a context-aware manner. Safety is the most important concern in maritime traffic control, and special care must be taken to avoid collisions between vessels at sea. It presents an architecture for an adaptive information visualization system for a maritime traffic control service. The proposed system adaptively determines the information to be displayed based on the safety evaluation scores and expertise of vessel traffic service operators. It also introduces a method for safety context acquisition to assess the risk of collisions between vessels, using parallel and distributed processing of maritime stream data transmitted by sensors on the vessels at sea. It provides an information-filtering, knowledge extraction method based on the work logs of traffic service operators, using a machine learning technique to generate a decision tree. We applied the proposed system architecture to a large dataset collected at a port. Our results indicate that the proposed system can adaptively select traffic information according to port conditions and to ensure safety and efficiency.

## 1. Introduction

With advances in sensor technology and communication infrastructure, increasing amounts of measurement data are generated and collected using sensors. In monitoring services, these data must be displayed on screens for operators [1]. The limited size of display screens does not allow for all information to be displayed, given the large volume of data collected. In most situations, all of the available information is not useful for the operators of monitoring services. This type of information overload distracts operators because they are forced to filter unnecessary information to find relevant and useful information. The mental burden incurred by this filtering leads operators to make mistakes. Because mistakes in monitoring services can be catastrophic, it is important to display only contextually meaningful information to monitoring service operators. 

Maritime traffic monitoring is a crucial service in maritime transportation because of the following unique characteristics: (1) in contrast with roads and highways, there are no visible paths at sea. (2) Sea vessels vary in size and communication capabilities [2]. (3) Vessel movements are highly inertial; it is difficult for vessels at sea to rapidly change course and accelerate or decelerate. Therefore, it is necessary to provide proper traffic control services to prevent vessels from colliding with other vessels or obstacles. In the literature, there have been some works for visualizing maritime traffic information [3].

In harbors and coastal waters, vessel traffic service (VTS) stations monitor vessel traffic to enhance safety and efficiency and preserve the maritime environment [4]. Most vessels are equipped with various sensors to measure their status, movements, and environment. Because of wear and the poor maintenance of these sensors, as well as environmental disturbances, measurement data from vessel sensors are prone to corruption and duplication. VTS operators monitor vessel traffic on screens that show vessel statuses at any given time, and this information may be contaminated by sensor noise. In particular, when the number of vessels is large in a given area, it is stressful for a VTS operator to coordinate traffic without error. Some information systems have been developed to assist operators in understanding and effectively handling scenarios involving heavy traffic, predicting the courses of vessels, and conveying instructions to navigators. While such systems have improved over the years, they are not yet sufficiently functional to relieve operators of their monitoring responsibilities altogether. Human agents are needed to examine specific situations, using such tools as risk evaluators to make critical decisions. 

**Figure 1:**
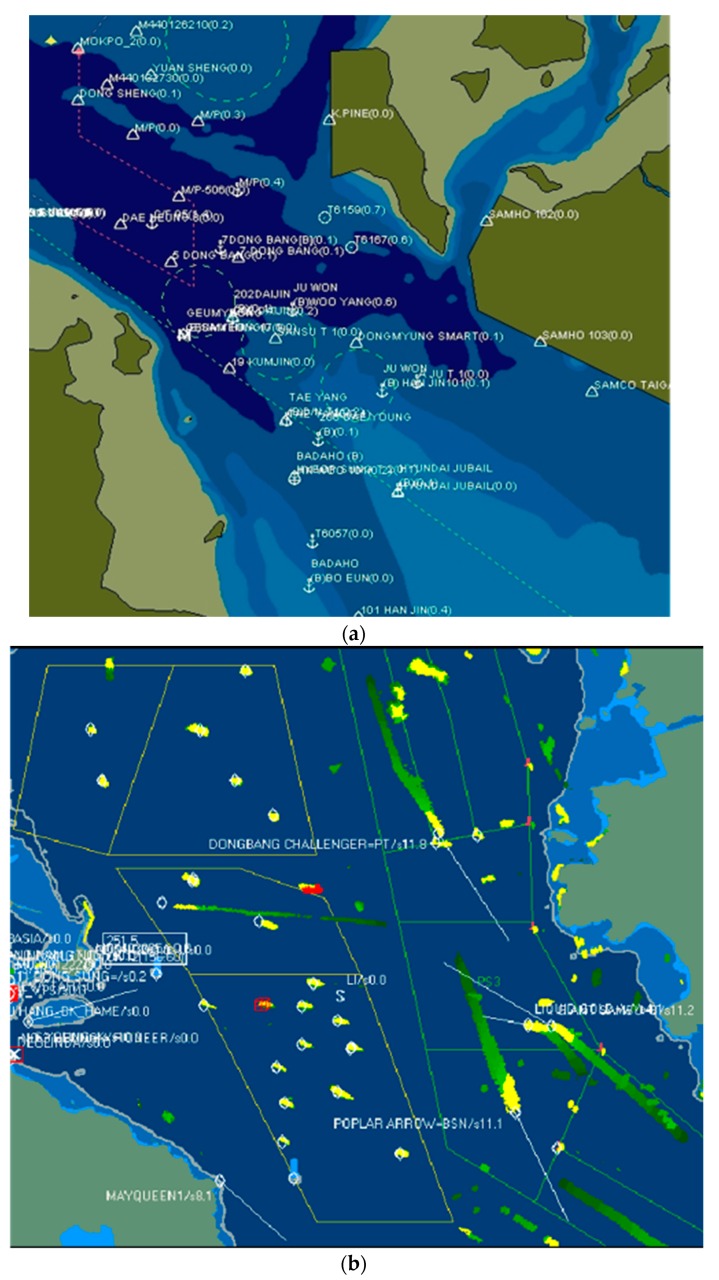
(**a**) ECDIS screen illustrating information overload. (**b**) Limited information that is easier to understand.

Current maritime traffic systems do not address certain problems. They do not effectively manage information overload, whereby all available information concerning all vessels is displayed on a monitoring screen, called an electronic chart display and information system (ECDIS). As the number of vessels increases, the information items tagged to the icon of a given vessel on a screen begin to overlap, making it challenging for operators to extract meaningful information from a display. The ECDIS allows operators to manually remove certain information. Figure 1a shows a monitoring screen displaying vessels and their associated information. Figure 1b shows that some irrelevant information is ignored. Vessel information includes the call sign, specifications, navigation status, schedule, and indications relating to violations of regulations. When operators manually turn off, resize, or change the colors of information items, they may have to weigh their importance in terms of safety and efficiency. Safety is the most important concern of monitoring services. In conventional monitoring information systems, the risk of collision is evaluated by examining the pairwise projective courses of manually selected vessels.

A large number of vessels are present in harbors and coastal waters at any given time, and the information pertaining to these vessels dynamically changes. These changes are measured by sensors and delivered to control services by wireless networks in the form of stream data. Because of the duplication and corruption of data, some preprocessing is needed to clean these data. Stream data consist of ordered data items collected continuously. Vessel monitoring services should be provided in real time over large areas. Traditional information systems struggle to provide advanced services and require heavy computation because of time constraints. This paper presents a distributed parallel processing method that preprocesses input stream data in order to extract contextual information, such as the risk of collision. 

It is burdensome for operators to adjust manually the appearance of information items in dynamic situations because vessels constantly enter and exit the monitoring region [5]. The information overload on the operators could be mitigated by the computing system that adjusts automatically the appearance of information items in a manner similar to expert human operators. Operators make judgments by recognizing the situational context and using their experience. To build such a system, it is required to have some methods to acquire contextual information and to extract knowledge from domain experts like VTS operators. 

In this paper, we propose a prototype architecture designed to extract contextual information from the sensor stream data of vessels as well as pilot and port management information systems. Our paper also presents a machine learning-based method to extract expert knowledge from the work histories of operators. Machine learning is a set of techniques that can automatically extract patterns or regularities from a collection of data. The proposed method learns from work history data and constructs a decision tree concerning the information to be displayed in the recognized context. 

The remainder of the paper is organized as follows: Section 2 provides background information and related work on maritime monitoring services, collision risk assessment, and operator information overload. Section 3 describes the proposed system architecture, the parallel distributed processing method for context recognition (which requires heavy computation), the context extraction method, and the knowledge extraction method for handling information overload. Section 4 explains our experimental settings and results, and Section 5 summarizes our conclusions.

## 2. Background and Related Work 

### 2.1. Maritime Monitoring Service 

There have been some web-based vessel monitoring service websites [6] that allow to track vessels worldwide. They provide some pieces of such basic information about vessels as vessel status and collision risk index. Their web interface facilitates the operations of zooming-in and zooming-out of the view, in which zooming-in makes detailed information displayed on the screen and zooming-out causes only abstract information to be displayed on the screen. The amount and kind of information items are limited which are to be displayed on their screen. They have no such serious issues of information overload as in VTS monitoring service systems.

**Figure 2:**
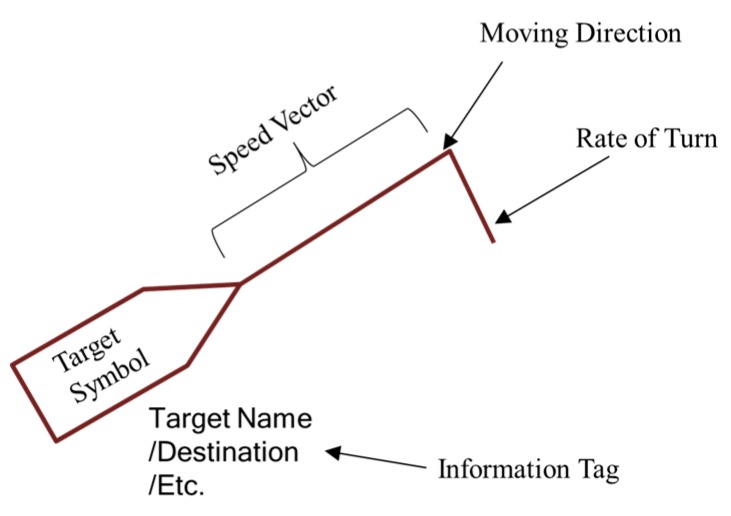
Symbol annotation used in ECDIS.

Maritime traffic service systems, such as VTS systems, conventionally use the symbol shown in Figure 2 to indicate the navigation state of a vessel. The center coordinate of such a symbol indicates the location of the GPS sensor position of the target vessel. The stretched line segment indicates the vessels’ speed vectors extending in the direction of her course of ground (COG), and the last line segment indicates the vessel’s rate of turn. Additional information about the ship, such as ship information, navigational status, pilot embarkation, risk of collision, and regulation violations, is tagged below the symbol [7]. Some information is available directly from the system whereas other items, such as the risk of collision and pilot embarkation estimations, must be calculated at the demand of users. 

When all available information is displayed, an ECDIS screen appears messy and crowded, and users can become fatigued when searching for and discriminating between the required information. “Information overload” refers to conditions wherein excessive information items are displayed. Information overload reduces job satisfaction and worker productivity and may cause serious damage, including loss of life, if operators make errors such as overlooking potential accidents.

Figure 1 illustrates a case of information overload as well as a case where information is easier to understand. If an information system is in a “friendly” state, it is good for user service. However, if the system is in a state of information overload, users must manage the complexity and information surpluses by turning off displays of unnecessary information. If the entities displayed are not dynamic, this type of manual manipulation is not difficult. However, in a monitoring system, entities constantly appear and disappear. Therefore, manual adjustment becomes challenging for operators and may lead to significant problems. 

To control the display of information items, Kim and Lee [8] proposed a context-aware rule-based method. Their method is supposed to build manually such rules by asking the VTS operators questionnaires. The performance of this method, therefore, strongly relies on the quality of the questionnaires made and the VTS operators’ expertise who answer the questionnaires. 

This paper proposes an adaptive information visualization method for mitigating information overload by automatically adding or removing information items from a display screen for vessel traffic control. The proposed method uses a machine learning technique for automatically extracting from expert work histories (i.e., VTS operator work logs) the knowledge of controlling the display of information items. It also has an architecture for recognizing the operational contexts from stream sensor data. 

### 2.2. Collision Risk Evaluation

**Figure 3:**
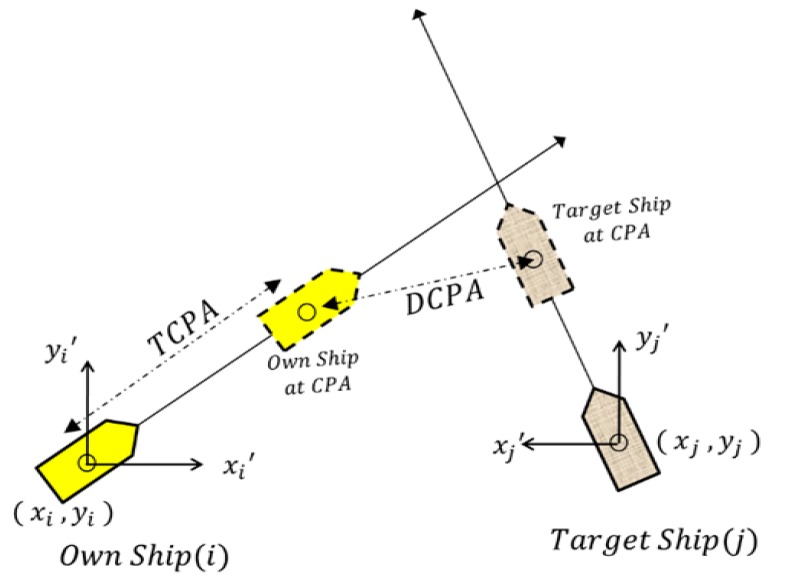
Evaluation of ship collision risk using DCPA and TCPA.

In maritime monitoring services, VTS operators focus heavily on the safety of vessels and facilities in areas under their charge, such as harbors and coastal waters. Because of the nature of vessel dynamics, vessels rarely change course drastically. Thus, if the near-future trajectories of two vessels are evaluated, it is possible to estimate their collision risk by computing the distance at the closet point of approach (DCPA) and the time to the closest point of approach (TCPA). The DCPA is the distance between vessels that make their closest approach. The TCPA is the amount of remaining time until two vessels reach their closest point. Figure 3 represents evaluation of ship collision risk using DCPA and TCPA.

TCPA and DCPA are computed using the following Formula [9].

(1) $$\mathrm{TCPA}=\frac{-\left[\Delta y\left(v_{t}sin\theta_{t}-v_{o}sin\theta_{o}\right)+\Delta x\left(v_{t}cos\theta_{t}-v_{o}cos\theta_{o}\right)\right]}{{\left(v_{t}sin\theta_{t}-v_{o}sin\theta_{o}\right)}^{2}-{\left(v_{t}cos\theta_{t}-v_{o}cos\theta_{o}\right)}^{2}}$$

(2) $$\mathrm{DCPA}=\sqrt{\begin{array}{c} {\left[\Delta y+\left(v_{t}sin\theta_{t}-v_{o}sin\theta_{o}\right)\times TCPA\right]}^{2}+\\ {\left[\Delta x+\left(v_{t}cos\theta_{t}-v_{o}cos\theta_{o}\right)\times TCPA\right]}^{2} \end{array}}$$

here, the own vessel and the target vessel position coordinates are denoted by ($x_{o},y_{o}$) and ($x_{t},y_{t}$) respectively. $v_{o}$ and $v_{t}$ are the own and target vessel speeds, respectively. $\theta_{o}$ and $\theta_{t}$ are the own and target vessel courses, respectively.

Most maritime monitoring information systems provide functionality of computing collision risks between selected vessels. Several methods have been proposed for evaluating a measure called the collision risk index, such as DCPA, TCPA and relative bearing change. A widely used collision index is the encounter risk indicator $F_{st}$, which is defined with respect to the CPA and TCPA as follows [10]:

(3) $$F_{st}=\;e^{-\left|CPA\right|}\cdot e^{-6TCPA}$$

Two vessels located within a certain range of a crossing region are called encountering vessels. The encounter risk indicator simply combines the CPA and TCPA as shown in (3), which yields a value in the interval [0,1]. 

Mou et al. [11] developed a dynamic risk model using the CPA, TCPA, and encounter angle between vessels. Kim et al. [12] introduced a logistic regression method for evaluating near-miss collisions. Hasegawa [13] proposed a fuzzy logic-based method combining data from the DCPA and TCPA with fuzzy rules to estimate the collision risk index. Hammer et al. [14] developed a collision risk estimation method reflecting the relative distance and relative angular velocity of one vessel to another. 

Generally, monitoring information systems do not compute the collision risks of all possible pairs of vessels because of the real-time constraints of having to display the given states of traffic. Moreover, even though monitoring systems can compute such indexes, it is too messy to display all computed pairwise indexes on a screen. 

### 2.3. Information Overload

When the magnitude of inputs becomes too large for users to process, information overload occurs. The management of information overload is crucial in both monitoring services and process-based service domains where appropriate human involvement is required [15,16]. Various countermeasures are available for mitigating information overload with respect to operator burden, including task and process parameters, organizational design, and information technology applications. Countermeasures pertaining to the operators include training programs for augmenting information literacy, systematic priority management training, and training to improve information screening skills [17]. Task and process parameter-based countermeasures include the standardization of operating procedures, the establishment of decision models in specific decision processes, and the regulation of information flow rates. Organizational design-based countermeasures include the organization of lateral relationships for backups, the reduction of individuals’ divergence through socialization, and the engagement of additional workers. Information technology applications-based countermeasures include the provision of information by pushing rather than pulling technologies, the automatic prioritization of information using mechanisms like voting, expert committees, and the provision of intelligent user interfaces that inform users of important problems and associated candidate solutions. 

The adaptive information visualization addressed in this paper belongs to the category of information technology applications-based countermeasures. Intelligent information provision can reduce information overload and can lessen human errors and improve productivity. From an information technology perspective, a fundamental countermeasure for mitigating information overload is filtering out information items that are not required at a given time. Rather than paying attention only to relevant information, operators working under information overload expend effort to identify and ignore irrelevant items. Therefore, information systems, especially those used for monitoring work, should suppress unnecessary information. Decisions about whether specific information is needed at a specific moment are made by operators who are overloaded with information. Therefore, an important source for acquiring filtering knowledge is data from the work histories of operators using information systems. It is promising to apply a machine learning algorithm to extract knowledge about filtering operations from a collection of work histories. The proposed method uses a machine learning algorithm to extract this knowledge, with the goal of selecting which information should be displayed. A decision tree algorithm is used to build this filtered knowledge. 

## 3. Adaptive Information Visualization Method for Maritime Traffic Stream Data 

### 3.1. Architecture of Adaptive Information Visualization System

Monitoring service systems should mitigate information overload to enhance worker satisfaction and performance. A useful approach for filtering out irrelevant information is to simulate the behavior of expert users to mitigate information overload. Using the work histories of expert users as a data source, machine learning algorithms can extract expert decision knowledge. Expert users analyze situations and make decisions based on the results. The results of these situational analyses can be denoted based on context. Expert users recognize context and, accordingly, make decisions based on expertise. 

**Figure 4:**
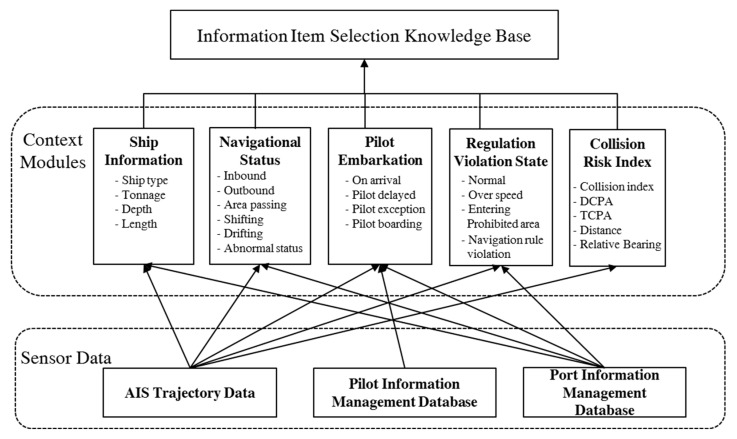
System architecture for adaptive information visualization in maritime traffic monitoring.

In the maritime monitoring service system, we use an approach similar to that taken by experts. Figure 4 shows the system architecture of adaptive information visualization in maritime traffic monitoring. The architecture consists of three layers: a sensor data layer, a context module layer, and an information item selection layer.

The sensor data layer indicates the location of data sources such as automatic identification system (AIS), trajectory data, pilot information management data, and port information management data. VTS operators use these datasets to make decisions about which information to display on their monitoring screens. The AIS is an automated vessel tracking system that displays other vessels in the vicinity. To facilitate AIS services, vessels periodically broadcast their information, including ship name, call sign, longitude, latitude, course, speed, direction, rate of turn, time, ship type, ship length, and ship width, to other vessels nearby. The following is an example of an AIS message:


             !AIVDM,1,1,,A,16ShEK0P0795K6TC9sh=Q?wn0HRt,0*23


AIS messages like the above are parsed and their constituents are coded by other vessels AIS systems. Such messages are generated every three seconds to three minutes, depending on the vessel speed and course changes [18]. VTS stations receive all AIS messages in their areas of coverage and keep track of vessels to control maritime traffic. 

Pilot information management data consists of the pilot embarkation and disembarkation schedules, pilotage passage, ship information, and pilotage stage. Maritime pilots are professional sailors who maneuver vessels through congested and dangerous waters such as the water near harbors. The pilotage areas of vessels are important in maritime traffic control. 

Port information management data are records of the movements of ships and cargo in and out of ports. Using these data, stakeholders can monitor vessels and cargo operations in a port in real time. The governing body of a port can use this dataset to compute usage fees for a port and its facilities. A statistical analysis of the dataset allows for the extraction of statistics concerning port operations, such as vessel entries and departures, usage of port facilities, and cargo volume. This dataset is a valuable source for examining efficient port operations. 

The context module layer consists of the following context extraction modules: ship information, navigation status, pilot embarkation, compliance with regulations, and collision risk index. Contextual information usually consists of the results of raw data processing. Ship information comprises information on the ship type, tonnage, depth, and length. The navigation status of a ship is obtained by analyzing its position, speed, and direction as well as its port information management data. The pilot embarkation status is obtained by analyzing pilot information management data. The state of regulation violations is determined by reviewing maritime regulations publications and comparing those regulations against a vessel’s position. The collision risk index is computed by the techniques described in Section 2.2. This contextual information corresponds to context recognition by an expert as the proposed method extracts such information that supports decision making. 

The top layer is the information item selection knowledge base, which is used to select the information items that will be displayed. This filtering is done to manage information overload and effectively provide necessary information to users. The knowledge base was constructed using machine learning to simulate the decision logic of experienced VTS operators. To this end, raw data regarding VTS operator decisions were recorded, and the former was transformed into contextual data using context extraction methods. Therefore, the training data consisted of contextual information and associated VTS display information control by VTS operator decisions.

### 3.2. Parallel and Distributed Evaluation of Collision Risk and Estimating Pilot Embarkation 

In maritime traffic control, the safety of vessels is evaluated by collision risk indexes, as shown in Equation (3). The collision risk index is calculated by pairwise comparisons of the own and target vessels’ DCPA and TCPA data, which means near-future closest encounter situation. These calculations may require massive amounts of computation as the number of candidate pairs increases quadratically with the number of vessels [19]. In monitoring services, these computations should be accomplished in real time [20]. The monitoring system should be scalable in terms of the monitored vessels and areas. 

**Figure 5:**
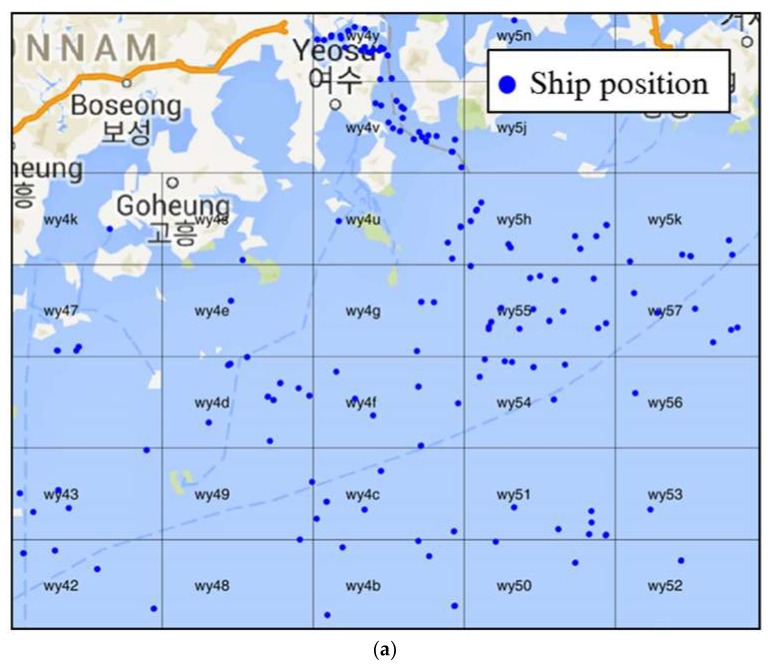

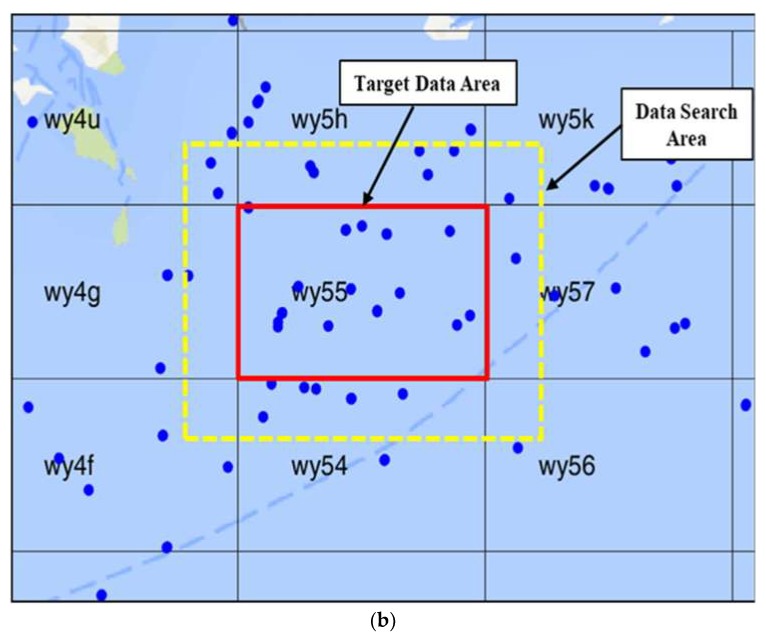
(**a**) Grid-based indexing of vessel locations. (**b**) Target area in a grid block and data search area.

When evaluating collision risks for pairs of vessels, some pairs do not have to be examined because the likelihood of collision is very small. For selecting candidate pairs, we investigated indexing data structures like the R tree [21], PPR tree [22], and R * tree [23]. These data structures are efficient for entities that are stationary or nearly stationary, but the management cost is high in maritime traffic monitoring services in which vessels keep moving for a majority of their time. Thus, the proposed method does not use such tree-based indexing structures, and instead uses a grid-based indexing structure, as shown in Figure 5. 

The grid-based indexing structure is organized according to Geohash [24], which hierarchically divides the globe into a grid and encodes each grid cell with a string. A grid can be considered to be a longitude–latitude rectangle, and a z-order traversal covers the globe at each resolution. The neighboring blocks of a grid can be determined from its Geohash value. As the length of the Geohash string increases, the resolution becomes finer. The levels of resolution range from 1–12, with higher levels indicating smaller regions. At level 8, a grid covers a region of size 38.2 × 19.1 m, which is small enough to specify the location of a vessel. Thus, a level 8 Geohash is sufficient for encoding the longitude–latitude locations of vessels. To assess collision risk, the proposed method divides the globe into level 5 Geohash grids, each of size ≤4.89 × 4.89 km. When the collision risk of a vessel in a level of the Geohash grid (i.e., the target data area) is evaluated, other vessels are at the same level of the grid or an adjacent level, as shown in Figure 5b (i.e., the data search area). When a vessel is located near the boarders of a grid block, the collision risk index module searches for data in the data search area as shown in Figure 5b. Once some vessels are found in the data search area, the pairwise collision risk for all vessels in the area are additionally evaluated. 

**Figure 6:**
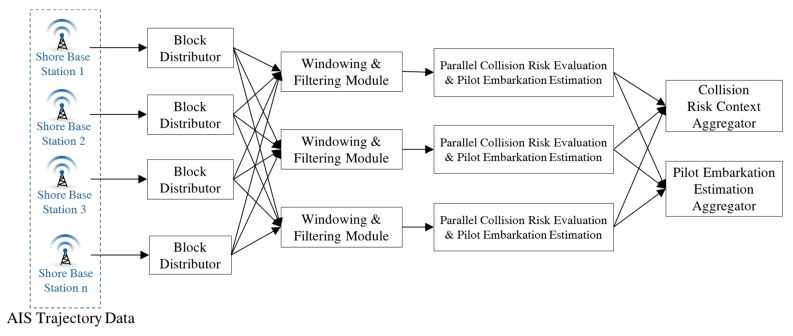
Parallel distributed processing for assessing collision risk and estimating pilot embarkation.

For scalability against time constraints in monitoring services, the proposed method uses a parallel distributed processing architecture to assess the risk of collision and estimate pilot embarkation, as shown in Figure 6. Each grid block can be processed on a different computing server; however, servers can treat multiple blocks because they possess sufficient resources to handle them. AIS messages are collected at shore base stations, such as VTS stations. The collected messages are distributed to their corresponding computing servers because each grid block is assigned to a specific server earlier on. The data is distributed by a block distributor that delivers each AIS message to the corresponding server. For example, when an AIS message is received from a level 6 grid, it is delivered to the server for the level 5 grid. 

AIS messages have a timestamp and are periodically generated by each vessel. The monitoring system assessed collision risks at fixed intervals, typically every six seconds. During this time, some vessels may not send AIS messages [25]. Therefore, each time an AIS message is received, the existing AIS message for the same vessel is replaced by a new one. In Figure 6, a windowing and filtering module handles such data manipulations. A parallel collision risk evaluation and pilot embarkation estimation module computes the collision risk index for possible encounters of vessel pairs. This index is a value in the interval [0,1]. For each vessel, the module also extracts relevant pilot embarkation information, such as harbor pilot embarkation and disembarkation times, pilotage areas, assistant tugs, and pilot names. The modules are executed in parallel and independently. This allows the time constraints to be maintained by increasing the number of servers and distributing the operations over them. The outcomes of the modules are sent to a collision risk context aggregator and a pilot embarkation estimation aggregator. The collision risk context aggregator collects and maintains collision risk indexes from the distributed computing servers, and the pilot embarkation estimation aggregator collects corresponding information from servers. 

### 3.3. Contextual Information Extraction 

**Figure 7:**
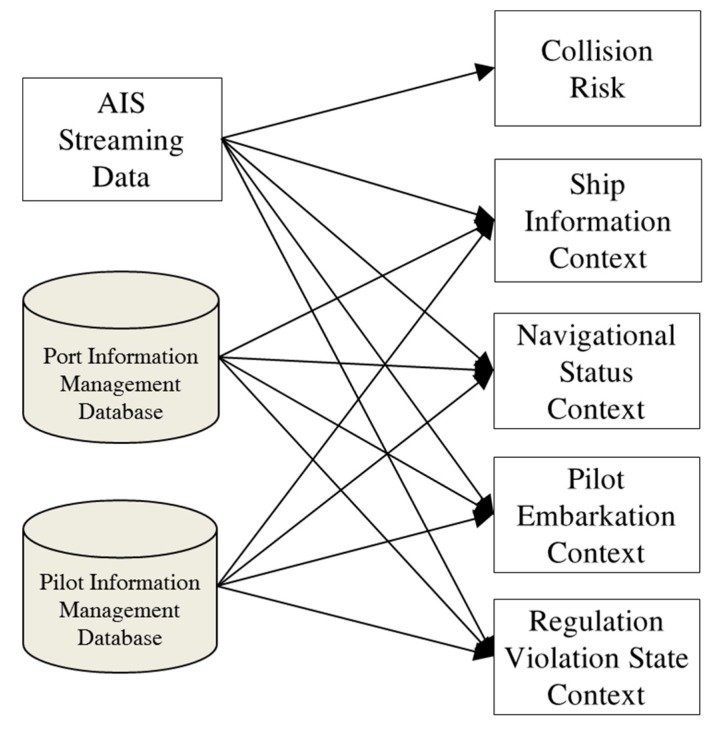
Context extraction modules for decision making.

The proposed system architecture for adaptive visualization contains the context extraction layer shown in Figure 7. The collision risk context module is constructed using the methods explained in Section 3.2. Context modules for ship information, navigation status context, pilot embarkation context, and state of regulation violations have been developed based on the knowledge of maritime traffic control.

The ship information context module is responsible for extracting call signs from AIS messages. The module also extracts ship type, tonnage, ship depth, and ship length from the port information management system.

**Figure 8:**
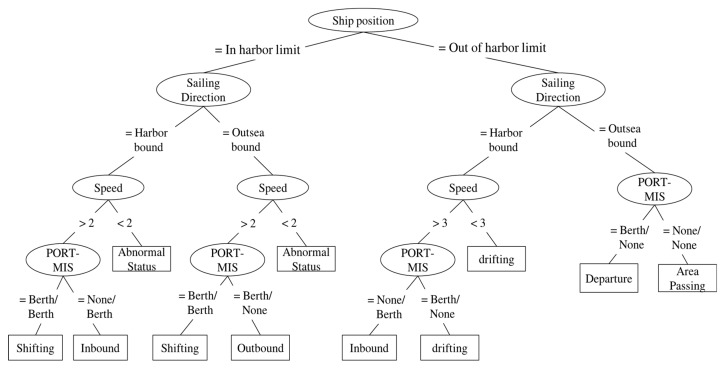
Knowledge used in the navigational status context module.

The navigation status context module classifies the navigation status of a vessel into one of the following states: “inbound,” “outbound,” “shifting,” “area passing,” “abnormal status,” or “drifting.” Figure 8 shows the classification knowledge expressed in a decision tree built with the help of domain experts, i.e., VTS operators. 

The pilot embarkation context module classifies the pilotage context of a vessel into one of the following states: “pilot board on arrival,” “pilot delayed,” “pilot exception,” “pilot onboard,” or “pilot disembarkation.” This classification knowledge has been extracted by knowledge engineering from domain experts. 

Regulation violation state context is determined by checking published regulations against a vessel’s current state. Its state belongs to one of the following categories: “normal state,” “over speed,” “entering prohibited area,” “navigation rule violation,” and “navigation rule violation with over speed.” 

### 3.4. Machine Learning-Based Knowledge Extraction for Information Overload Handling

In maritime traffic monitoring services, VTS operators suffer from information overload induced by the number of information items tagged for each vessel [26]. Table 1 presents the information items displayed on the screen of a maritime traffic control system. In conventional information systems, VTS operators manually choose to display each item of information. 

To automatically turn information items on or off on a monitoring screen, we use a machine learning algorithm that extracts knowledge from a training dataset. Human operators determine the recognized contexts, as explained in Section 3.3. We collected work histories of VTS operators by logging their actions concerning display items, along with the associated AIS data, pilot data, and port management data. In the experiments, the collected raw data have the following structure: the data above <Displayed Items> is the raw data, and the data below <Displayed Items> indicates the displayed items according to operator decisions.


<AIS trajectory data> 
Ship name: VINUS *****, Position: (34°38′55″N , 127°55′02″E) , Course: 330°, Speed: 13 knots,  rate of turn: 0.2°/min , call sign: DS***, time: 2017/06/01 10:15:00
<Port information management database>
Ship type : tanker, Ship size : 250m, draught : 13 m, last pier code: MBN-01, last pier departure time: 2016/05/21 10:05:00, next pier code: MBN-01, next pier estimation arrival time: 2017/06/01 12:00:00, event category: entrance
<Pilot information management database>
Pilot station : No.1 pilot station, onboard time: 2016/06/01 10:00:00, pilot ladder: portside 3m, pilot name: SK, pilot disembarkation: MBN-01
<**Displayed Items**> 
Ship status: position, ship name, course, speed, ship type, call sign
Destination: next pier
Collision: collision index, DCPA, TCPA
Pilot information: pilot name
Regulation violation: regulation violation information


Because operators make decisions based on recognized contexts, we transformed the raw data into contextual data using the context extraction modules described in Section 3.3. 

		
<Contextual data>
Ship Information: large size tanker, Navigational Status: inbound,  
 Pilot Embarkation: pilot onboard, Violation State: over speed, Collision Risk Index: 0.6
<Displayed Items> 
Ship status: position, ship name, course, speed, ship type, call sign
Destination: next pier
Collision: collision index, DCPA, TCPA
Pilot information: pilot name
Regulation violation: regulation violation information


To extract knowledge concerning decisions regarding which items of information to display, we applied the decision tree algorithm C4.5 [27], which extracts a decision tree from a collection of datasets. The decision tree algorithm organizes a tree-structured classification that concisely and accurately describes the training data. The algorithm tries to choose attributes that maximize information gain *I* (D, a) which is the difference between the average entropy avgE(D,a) of the partitioned results by the given attribute *a* and the entropy *E*(D) of data prior to the partition:

*I*(*D*, *a*) = *E*(*D*) *− avgE*(*D*, *a*) (4)

This algorithm is applied to each vessel individually whenever the contextual data changes. Even if the contextual data of a vessel data remains unchanged, the proposed method is executed at intervals set by the user (for instance, 10 s in a navigation situation and 30 s in an anchoring situation). Figure 8 shows a snippet of knowledge relating to turning the items of information on a screen on or off to reduce information overload, as constructed by the C4.5 algorithm.

## 4. Experiments

**Figure 9:**
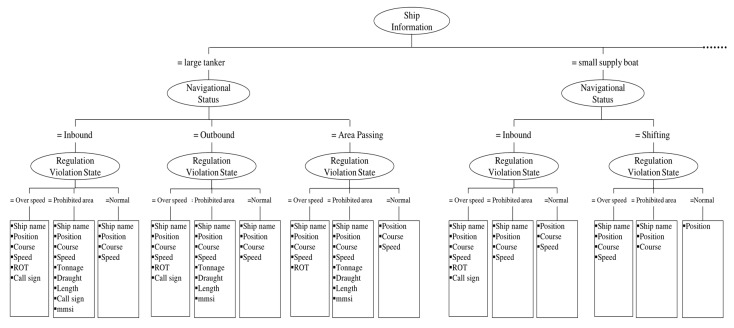
Snippet of the knowledge used to determine items of information to be displayed in a decision tree.

**Figure 10:**
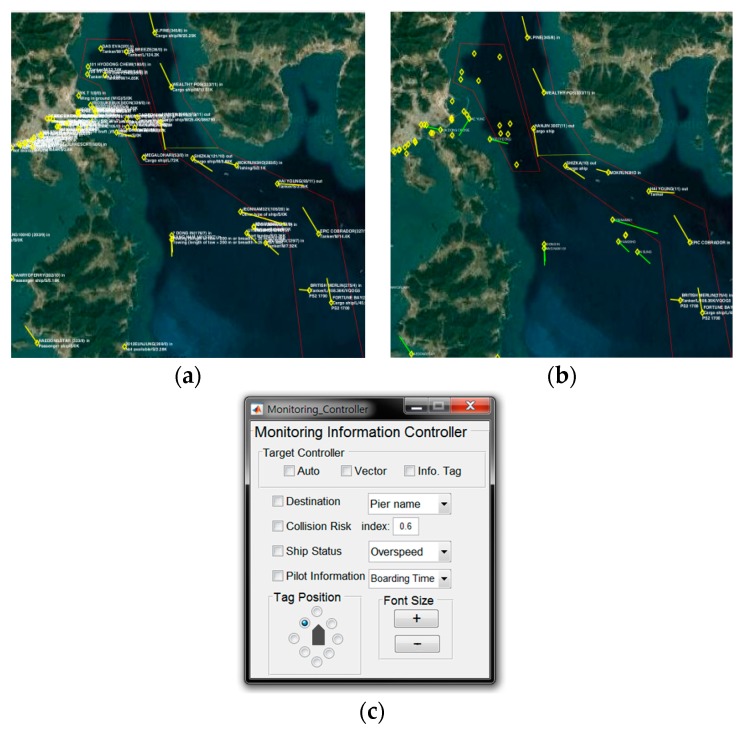
Screenshots of the prototype system. (**a**) A screenshot of the monitoring screen showing all information items. (**b**) A screenshot of the monitoring screen when the proposed method was applied. (**c**) The control panel used by the operator to adjust the displayed items.

To test the proposed method, we applied it to a dataset for the harbor at Yeosu in the Korean peninsula, as shown in Figure 9. We developed a dataset formulated over six months, containing 4.3 billion AIS messages from vessels visiting the harbor. Five months of data were used for training, with the remainder used for testing. Using the test dataset, we evaluated the extent to which the automatic information overload management system matched decisions made by VTS operators. The collision risk evaluation and pilot embarkation estimation modules were developed to be executed in a distributed manner. In the experiment, the area considered was relatively small. Therefore, six processes were executed and communicated via socket communication over two servers. These processes worked smoothly within the designed time constraints. We classified the vessels into three categories: inbound vessels for harbor, outbound vessels for harbor, and other vessels like pilot boats, tug boats, fishing boats which stay in the VTS monitoring area. In order to collect VTS information control log, we developed the prototype system as shown in Figure 10.

Table 2 presents our experimental results. In the experiments, the numbers of information items displayed by VTS operators were used as the reference, and they were compared to the numbers of information items displayed using a previous method (a rule-based information provisioning model) [8] and the numbers of information items displayed using the proposed machine learning-based model. The performance of the proposed method was measured by the relative difference ratio (RDR) defined as follows:

(5) $$RDR=\frac{NIM-NIV}{NIV}\times 100$$

here *NIM* indicates the number of information items displayed by the model and NIV is the number of information items displayed by the VTS operators. The objective of this work is to reduce the information overload of VTS operators by selecting automatically the information items to be displayed as if VTS operators do. The smaller the absolute value of RDR, the better the performance of the model. 

*Analysis of Vessel Status Information Selection*: VTS operators hide most information items on their display when a vessel arrives at the harbor. They show up important information items on their display when a vessel starts out outbound navigation from the harbor. Both the rule-based information provisioning system (RIPS) and the trained model by the proposed method (TM) detect the arrivals and the departures of vessels by their GPS data and speed. Both methods showed good performance for inbound and outbound vessels. TM is quite good as much as VTS operators. For the vessels of the other vessels category, their traffic patterns are not stationary because their speeds and courses are affected by their tasks and neighboring traffic conditions. This makes it difficult to extract the solid patterns for the other vessels category. TM did not display about 26.7% of the information items selected by VTS operators whereas RIPS did not display about 44.1% of them. It means that TM has made an improvement of 17.4% point over RIPS. 

*Analysis of Destination Information Selection*: For the inbound vessels, TM outperformed RIPS by 17.8% point. For the outbound vessels, TM outperforms RIPS by 10.8% point. The vessels in the other vessels category have no destination information item in their AIS data. Hence there are no experiment results for the category in Table 2. 

*Analysis of Collision Risk Index*: For the inbound vessels, RIPS displayed 4.1% point less information items than VTS operators whereas TM displayed 4.8% point less information items than VTS operators. For the outbound vessels, both RIPS and TM displayed a smaller number of information items than VTS operators by 3.2% point and 4.7% point, respectively. On the other hand, for the other vessels category, both methods showed much lesser number of information items than VTS operators while TM made 25.4% point improvement over RIPS. Vessels in the category move in the monitoring area in a somewhat unexpected manner. This makes it hard to construct manually the rules for displaying information items and for the employed machine learning technique to find sufficiently reliable rules.

*Analysis of Pilot Information Selection*: VTS operators pay more attention to pilot boarding area rather than pilot discharging area because pilots board on the vessels from a pilot boat on the pilot boarding area. Due to complicated traffic patterns in the pilot boarding area, the VTS operators display many information items whereas both RIPS and TM have missed considerable amount of information items by 42.7% point and 34.6% point, respectively. Both methods seem not to be sufficiently excellent to capture the knowledge of VTS operators. For pilot discharging area, the performance of RIPS was better than that of TM. By the way, VTS operators usually pay less attention to pilot discharging area than pilot boarding area. It is more important to increase the performance for pilot boarding information items rather than that for pilot discharging ones. 

*Analysis of Regulation Violations Information Selection*: For the over speed situations, it is clear what VTS operators look at. Both RIPS and TM showed good performance. For the navigation rules violations situations, however, there are various factors for VTS operators to take into account depending on the vessel encounter situations. TM showed big improvement over RIPS on the navigation rules violations situation by 28.4% point. 

For the test data of size 82.327, the TM model constructed by the proposed method showed the performance of RDR 11.0%, while RIPS showed the performance of RDR 18.1%. RIPS was constructed by questionnaire-based knowledge engineering for VTS operators. The experiments showed that the proposed machine learning-based method outperforms the questionnaire-based knowledge engineering approach in the VTS information overload problem. 

Even though the proposed method enables to get an improved model over the knowledge engineering approach, there is still some margin for improvement. While our method builds a single decision tree model, there have been reports that an ensemble of such decision trees may make an additional improvement. We expect such ensemble-based approach to build an improvement model. In the experiments, we used a training dataset of 5 months, and hence, it is expected model improvement can be achieved by using a training dataset over a longer period. The proposed method trains a model with a training dataset of a specific VTS monitoring area. Therefore, the trained model might as well reflect regional factors such as passage characteristics, vessel traffic, geographic characteristics, and so on. It means that a trained model for a VTS monitoring area may not work well for other VTS monitoring area. This machine learning approach requires to build its own model for each VTS monitoring area with its training dataset.

## 5. Conclusions

Information overload is a critical concern in traffic monitoring services, where conditions can change rapidly. The manual control of information overload sometimes requires extra work to extract information that helps decision making. Monitoring systems must provide services in real time. This additional filtering work distracts operators from their primary task of ensuring the safety of vessels at sea. 

In this study, we propose a system architecture for the adaptive visualization of monitoring services, which will automatically mitigate information overload on operators. The architecture consists of three layers. The bottom layer is the data source layer, which collects relevant data from multiple sources. The middle layer is the context extraction layer, which performs complicated operations to extract information supporting decision making. In monitoring services with strict time constraints, a parallel distributed processing architecture for Geohash-based partitions over the monitoring area can be successfully applied through scalability. We demonstrated the applicability of the proposed method within a maritime traffic control system. The top layer is the decision-making layer, which selects information items for display based on the states of all entities in the control area at the time. Information overload in monitoring services is handled by operators. A promising strategy for selecting which information to display is to mimic procedures adopted by field experts. 

The proposed system architecture for adaptive visualization can be applied to any monitoring service system with moving entities. The distributed parallel processing strategy for extracting intermediate results is particularly useful when a monitoring system has strict time constraints. 

## Figures and Tables

**Table 1:** Items of the information displayed in a maritime traffic control system.

Category	Information Items to Be Displayed
Ship status	Position, ship name, course, speed, rate of turn (ROT), ship type, ship length, ship width, tonnage, draught, nationality, call sign, MMSI, contact number
Destination	Last port, next port, next pier, last pier, estimated arrival time, cargo quantity, agent information
Collision risk index	Collision index, distance, relative bearing, DCPA, TCPA, CPA
Pilot Information	Pilot embarkation time, pilot disembarkation time, pilot station, assist tug, pilot name
Regulation violation	Regulation violation information

**Table 2:** Result of experiments.

Information Category	Monitoring Group	Number of Information Items Displayed by VTS Operators	Number of Information Items Displayed by the Models (RDR)	
			Rule-Based Information Provisioning Model [8]	Proposed Method
Vessel Status	Inbound Vessel	25,875	24,502 (▼5.3%)	24,731 (▼4.4%)
	Outbound Vessel	27,164	26,340 (▼3.0%)	26,684 (▼1.8%)
	Other Vessel	18,635	10,414 (▼44.1%)	13,667 (▼26.7%)
Destination	Inbound Vessel	4934	3779 (▼23.4%)	4648 (▼5.8%)
	Outbound Vessel	4555	3631 (▼20.3%)	4122 (▼9.5%)
Collision Risk Index	Inbound Vessel	2524	2421 (▼4.1%)	2402 (▼4.8%)
	Outbound Vessel	2423	2345 (▼3.2%)	2310 (▼4.7%)
	Other Vessel	5148	2341 (▼54.5%)	3651 (▼29.1%)
Pilot Information	Pilot Boarding	4876	2793 (▼42.7%)	3187 (▼34.6%)
	Pilot Discharging	2190	1898 (▼13.3%)	1872 (▼14.5%)
Regulation Violations	Over speed	1267	1208 (▼4.7%)	1247 (▼1.6%)
	Violations of Navigation Rules	954	655 (▼31.3%)	926 (▼2.9%)
Total		100,545	82,327 (▼18.1%)	89,447 (▼11.0%)
